# SIGMA: A System for Integrative Genomic Microarray Analysis of Cancer Genomes

**DOI:** 10.1186/1471-2164-7-324

**Published:** 2006-12-27

**Authors:** Raj Chari, William W Lockwood, Bradley P Coe, Anna Chu, Devon Macey, Andrew Thomson, Jonathan J Davies, Calum MacAulay, Wan L Lam

**Affiliations:** 1 Department of Cancer Genetics and Developmental Biology, British Columbia Cancer Research Centre, Vancouver, BC, Canada; 2 Department of Cancer Imaging, British Columbia Cancer Research Centre, Vancouver, BC, Canada; 3 BC Cancer Research Centre, 675 West 10th Avenue, Vancouver, BC, V5Z 1L3, Canada

## Abstract

**Background:**

The prevalence of high resolution profiling of genomes has created a need for the integrative analysis of information generated from multiple methodologies and platforms. Although the majority of data in the public domain are gene expression profiles, and expression analysis software are available, the increase of array CGH studies has enabled integration of high throughput genomic and gene expression datasets. However, tools for direct mining and analysis of array CGH data are limited. Hence, there is a great need for analytical and display software tailored to cross platform integrative analysis of cancer genomes.

**Results:**

We have created a user-friendly java application to facilitate sophisticated visualization and analysis such as cross-tumor and cross-platform comparisons. To demonstrate the utility of this software, we assembled array CGH data representing Affymetrix SNP chip, Stanford cDNA arrays and whole genome tiling path array platforms for cross comparison. This cancer genome database contains 267 profiles from commonly used cancer cell lines representing 14 different tissue types.

**Conclusion:**

In this study we have developed an application for the visualization and analysis of data from high resolution array CGH platforms that can be adapted for analysis of multiple types of high throughput genomic datasets. Furthermore, we invite researchers using array CGH technology to deposit both their raw and processed data, as this will be a continually expanding database of cancer genomes. This publicly available resource, the *System for Integrative Genomic Microarray Analysis *(*SIGMA*) of cancer genomes, can be accessed at .

## Background

Array comparative genomic hybridization (CGH) is a method used to detect segmental DNA copy number alterations and is widely used to discover chromosomal aberrations in cancer and other genetic diseases [1,2]. In this method, differentially labeled genomic DNA samples are competitively hybridized to chromosomal targets, and the copy number balance between the two samples is reflected by their signal intensity ratio. Numerous array CGH platforms exist; these vary in the type of elements present on the array and their corresponding coverage of the human genome. With the development of high resolution, genome wide arrays, tens of thousands of loci can be evaluated for copy number status, facilitating the high throughput search for genes potentially involved in pathogenesis. This has allowed the identification of discrete regions of alteration that may have been missed by traditional cytogenetic methods and has proven to be a useful platform for exploring the underlying genetic basis of cancer [1,3].

With the increasing utilization of array CGH, it has not only become important to establish standards for data deposition, but to develop tools to facilitate public access and to ease mining of available data. Currently, the *National Center for Biotechnology Information (NCBI) Gene Expression Omnibus (GEO) *repository [4] and *European Bioinformatics Institute (EBI) ArrayExpress *[5] provide storage for array CGH data, but these databases have been largely designed for gene expression microarrays. Although these sites support visualization of previously analyzed gene expression profiles, there are limited tools available for direct mining and analysis of array CGH data. Hence, there is a need for forums specific to array CGH data. Recently, attempts have been made in making a database primarily of lower resolution array CGH data [6]. However, with the accumulation of high density array data generated with diverse technology, the viewing of array data has become a bioinformatics challenge, especially when the integration of multiple datasets from different platforms is required. Therefore, a central database with analytical software tailored specifically for analyzing and visualizing different types of high resolution array CGH data would greatly facilitate data mining.

In this study we have created a database consisting of high-resolution, whole-genome array CGH profiles for nearly 200 commonly used cancer cell lines profiled on four different array platforms, which have been instrumental in biochemical and pharmacogenetic studies. Moreover, we have developed a user friendly, web-based java application called the System for Integrative Genomic Microarray Analysis (*SIGMA*) for comparative analysis of multiple genomes.

## Results and Discussion

### Cell-line collection

We have assembled a collection of 267 array CGH profiles, representing 184 distinct cell lines profiled on at least one of the four array CGH platforms (Table 1, Table 2). Moreover, 14 different cancer tissue origins and 30 distinct cancer types are represented in this database, resulting in the assembly of a wide spectrum of genomes in this repository (Table 2) [see Additional file 1]. Significantly, 56 of the 267 CGH profiles are unpublished raw data which is now made public.

### Main functionalities of SIGMA

**Figure 1:**
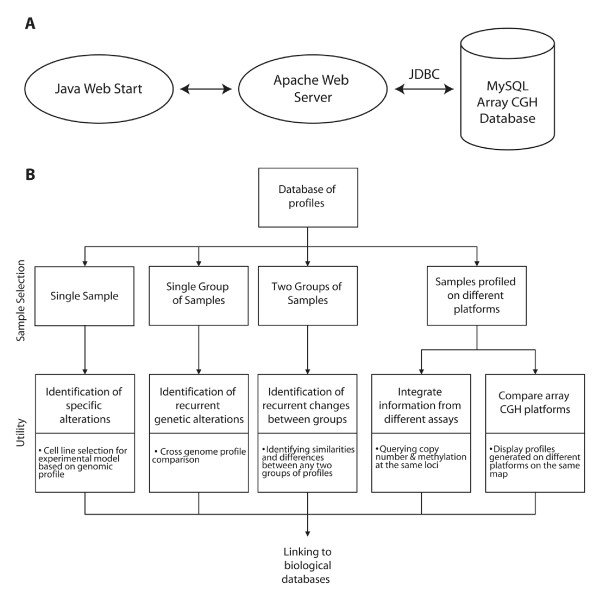
*(A) *Schematic of the basic architecture of *SIGMA*. *Java WebStart *technology was utilized to develop the main user interface to the database, ensuring users will be running the latest version of the program. The application is connected to the *MySQL *database through a JDBC driver provided by *MySQL*, over an Apache Web Server. *(B) *Outline of the various functionalities of *SIGMA*, with the five main types of visualization and analysis along with their associated uses.

In order to increase the utility of this collection, a significant component of *SIGMA *is the web-based application which allows for the user-friendly mining of this dataset. Four major types of functionalities are offered by *SIGMA*: (1) interrogation of a single sample, (2) visualization and analysis of a single group of samples, (3) comparative analysis of two groups of samples, and (4) integration of data from multiple platforms (Figure 1B).

### Visualization and interrogation of a single genome

**Figure 2:**
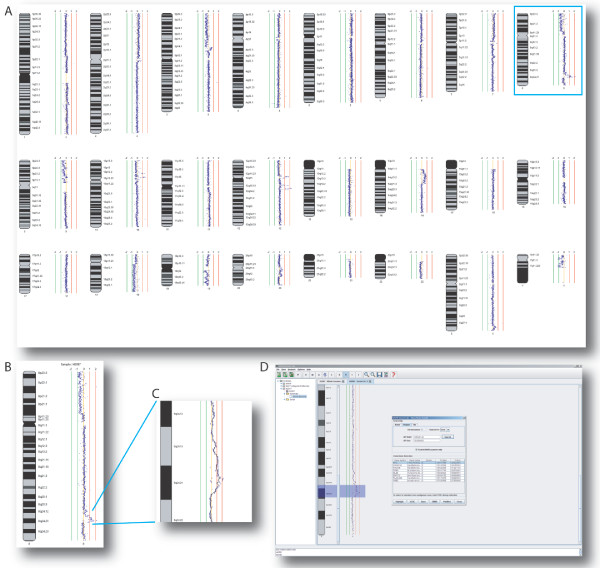
*(A) *Whole genome karyogram of the H2087 lung adenocarcinoma cell line profiled on the SMRT array using the May 2004 genomic build. For each chromosome, there is a ratio plot associated with plotting the log_2 _ratio of the genomic element vs. the position of the element on the chromosome. The log_2 _ratio of the data point is calculated against a normal reference where positive ratios represent increased content and negative ratios represent decreased content in the tumor compared to the normal. *(B) *The first level of zoom to view a particular chromosome, in this case, chromosome 8. *(C) *32X zoom into the amplicon at chromosome band 8q24.21. *(D) *The ability to link out to biological databases such as *NCBI MIM*, *UCSC Genome Browser*, *NCBI Gene *and *NCBI PubMed *with a gene of interest.

The first function we discuss is the ability to view a single high resolution array CGH profile at multiple magnification levels. The major utility of this function is to display the underlying genomic architecture of a cell line, so that genetic features can be considered in experimental interpretation. For example, a whole genome karyogram of lung adenocarcinoma cell line H2087 profiled on the SMRT array platform (Figure 2A). From this image, we see many changes such as the loss of the 3p arm as well as segmental changes in chromosomes 8, 19 and 20. Specifically, we can select chromosome 8 (Figure 2B) and view that separately, then zoom into the region of interest and visualize it in finer detail (Figure 2C). Users can then highlight or place boundary lines in this region and query for which genes are located within the region of interest.

Subsequently, using the interval search option, users can retrieve the genes which are located in a desired region and have the option to query commonly used biological databases such as *NCBI MIM*, *NCBI Gene*, *NCBI PubMed *and the *UCSC Genome Browser*. For example, if we look at band 8q24.21 (Figure 2D), we can highlight the region and search the interval for which genes it contains. When we invoke the region search and retrieve only genes curated by *RefSeq*, we see there are 8 genes in the amplicon. If the user selects a particular gene, options to link out to the above mentioned biological databases become available. The utility of this function is to facilitate a direct connection from experimental findings to known, relevant information. Moreover, the ability to interrogate for specific genes and regions can be done for any types of the analysis outlined (Figure 1B).

### Multiple genome comparison and mining across tumor types

**Figure 3:**
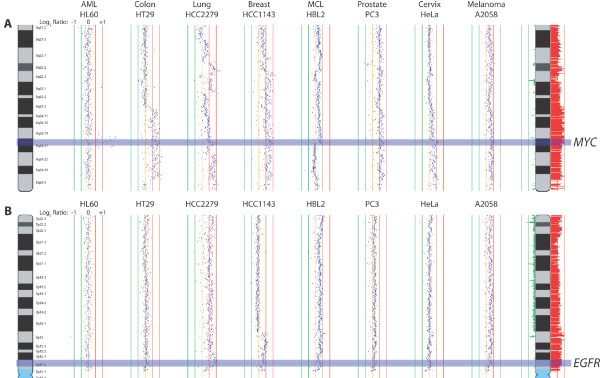
Serial view of 8 samples representing 8 different tumor types, demonstrating the breadth of data available. From left to right: HL60, HT29, HCC2279, HCC1143, HBL2, PC3, HeLa and A2058. *(A) *Chromosome 8, highlighting the amplification of the *MYC *oncogene, which appears to be amplified in all 8 tumor types. *(B) *Chromosome 7, highlighting the *EGFR *locus, where it appears that the lung cancer line (HCC2279) and the breast cancer line (HCC1143) harbor the amplification of this gene. Vertical lines denote log_2 _signal ratios from -1 to +1 with copy number increases to the right and decreases to the left of 0. Each black dot represents a single BAC clone.

A common research question is to look across a series of samples with common phenotype to identify recurrent genetic changes, for example comparison of lung adenocarcinomas [see Additional file 2]. With the spectrum of samples warehoused in *SIGMA*, such a query can be performed across multiple cancer types. For example, the alignment of a set of samples representing 8 different cancer types revealed common amplification of the *MYC *oncogene locus (Figure 3A), while the epidermal growth factor receptor *(EGFR) *locus is amplified only in a subset of samples (Figure 3B).

Recurrent alterations detected in one group of samples can be compared against those in another group, for example, the comparison of lung squamous cell carcinoma (SqCC) with cervical SqCC [see Additional file 3]. The strategy for comparison, for example the overlay of frequency plots derived from two groups of profiles, has been described elsewhere [7,8].

### Simultaneous visualization of data from multiple platforms

**Figure 4:**
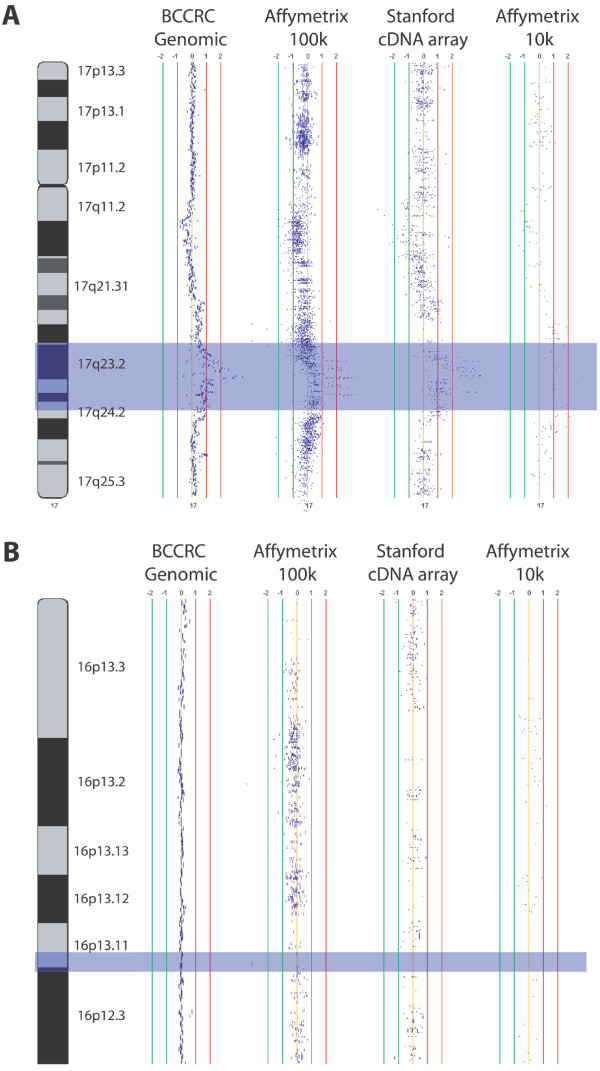
*(A) *Visualization of chromosome 17 of the breast cancer cell line MCF7 across multiple array CGH platforms using the May 2004 genomic build. The four platforms are labeled above each ratio plot. The architecture of the chromosome is consistent across all four platforms, with the altered region highlighted in yellow seen clearly across 3 of the 4 platforms. *(B) *Visualization of chromosome 16 of the same breast cancer cell line, illustrating decreased density of markers in 3 of the 4 array platforms, with the SMRT array providing greater coverage to a region centromeric to band 16p13.11.

Cross platform comparison is essential to the multi-dimensional descriptions of a genome. Here, we have included a feature in *SIGMA *to allow users to view multiple platforms of data simultaneously. We use the breast cancer cell line, MCF7, to illustrate this functionality. Data from four different array CGH platforms were available publicly: SMRT array, Stanford cDNA microarray, Affymetrix 10K SNP array and the Affymetrix 100K SNP array. Figure 4A illustrates the cross platform display of chromosome 17, while Figure 4B shows the variable density of coverage by these four commonly used platforms.

### Integrative visualization of DNA copy number and methylation

**Figure 5:**
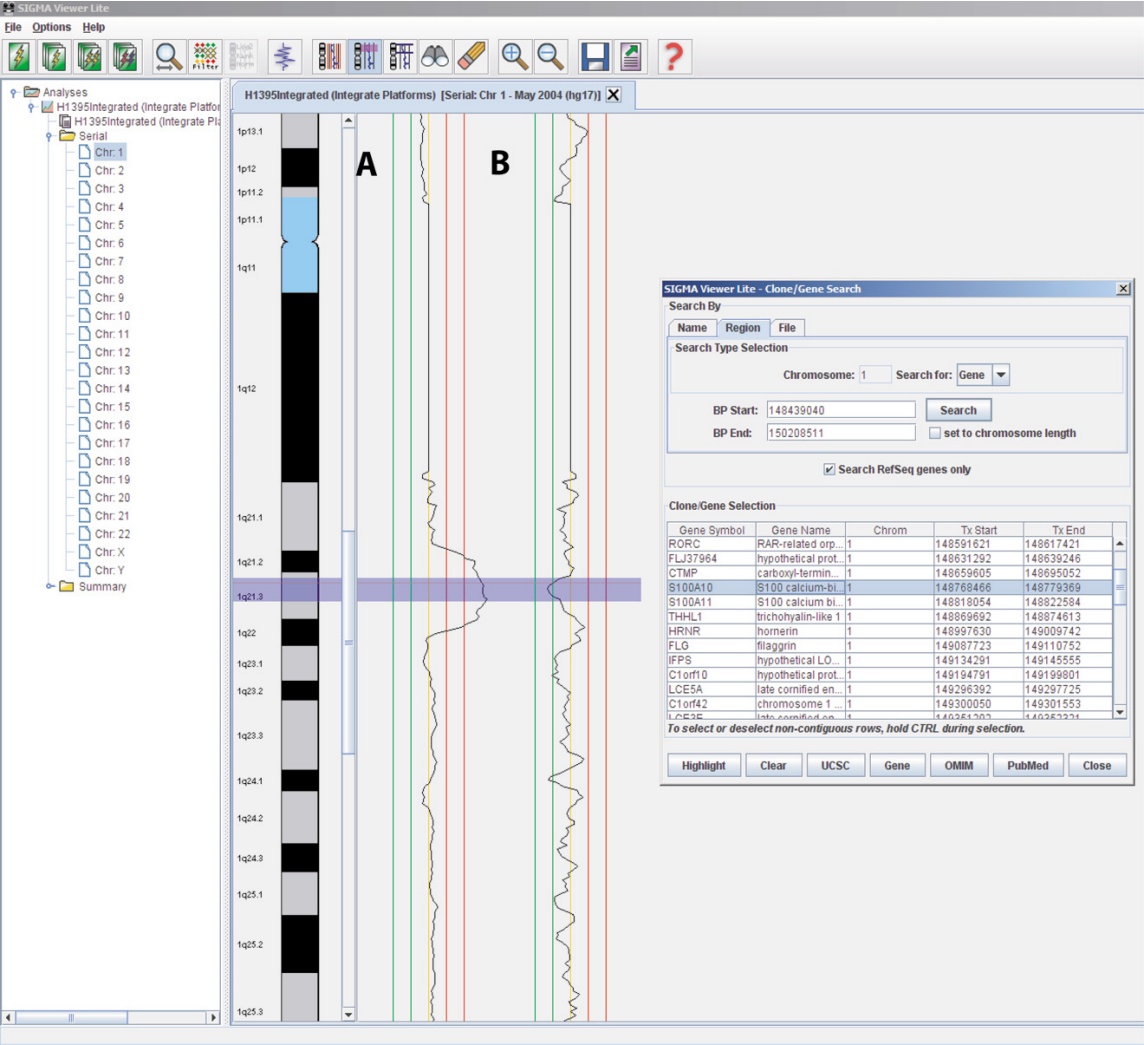
Integration of genomic and epigenetic profiles of the H1395 lung cancer cell line. *(A) *The genomic profile of H1395 is represented by a moving average spline of the data points. *(B) *Similarly, the epigenetic profile represents a residual plot of the tumor cell line subtracting the matching blood lymphocyte profile (BL1395), with a moving average spline representing the data points. The moving average was done at 1 MB intervals at increments of 200 kb for both profiles. Specifically, chromosomal region 1q21.3 is highlighted illustrating a region of both copy number increase and hypomethylation.

One of the novel features we have provided is the integrative visualization of copy number alterations with DNA methylation status. The major premise in studying copy number alterations at the DNA level is that these are the primary changes involved in driving changes in gene expression. Though gene dosage variation may be responsible for expression changes, alteration in DNA methylation pattern also contributes significantly to regulating gene expression. Recently, methods for global methylation analysis to measure aberrant DNA methylation status across tumor genomes have been developed [9-12]. Wilson *et al *(2006) compared methylation patterns with copy number profiles in lung cancer cells. Utilizing genomic and epigenetic data from this study for the H1395 lung cancer cell line, we illustrate a parallel display in *SIGMA*. Figure 5 shows a large segmental copy number gain spanning 1q21.2 to 1q23.1 with corresponding hypomethylation, localized precisely to 1q21.3 [13]. Significantly, both copy number gain and decreased methylation can elevate gene expression. The *S100 calcium binding protein A10 *(*S100A10*) gene within this region has been previously shown to be over-expressed in lung cancer [14]. It is readily apparent of the value of integrative studies examining aberrant DNA methylation and genomic copy number. With increased prevalence of studies of whole genome methylation, this feature will be of greater importance.

## Conclusion

We have developed an application for the integrative cross platform analysis of array CGH data. The *SIGMA *application facilitates consolidation and structuring of diverse sources of array CGH data into a repository that is accessible with a new easy-to-use built-in web-based analytical application. The launch version contains data for 267 array CGH profiles, representing cancer cell lines of over 14 different types of tissue. The ability of *SIGMA *to incorporate multiple array CGH platforms facilitates the archiving of array CGH data from future publications, regardless of current or future array platform used. Though currently *SIGMA*'s architecture facilitates the direct mining of genomic and epigenomic data, this can be easily adapted, and not limited to, high resolution genetic and gene expression surveys.

## Methods

### Sources of raw array CGH data

The raw data for 267 CGH profiles in the database was obtained from a variety of sources. They include both published and unpublished data [7,8,15-22]. Publicly available data were downloaded from *NCBI GEO *[4], *Stanford Microarray Database *[23] or websites affiliated with the author's laboratory. Data which were not publicly available were obtained by consent from the authors of the respective studies. The four array CGH platforms used for this study were the whole genome tiling path BAC (SMRT) array [24], the early access Affymetrix SNP 100 K array [16,20], the Stanford cDNA microarray [22] and the Affymetrix SNP 10 K array [15]. In addition, 2 of the cell lines were profiled for whole genome DNA methylation status using MeDIP array CGH [10,13]. For this launch version, we concentrated on available cell lines profiled on high density array platforms and did not include profiles from clinical specimens. A summary of the sources for the raw data is given in Table 1, while the detailed description of each of the cell lines in the collection is given in Additional file 1.

### Application layout of SIGMA

There are three main components which comprise this application; a *Java WebStart *application interface allowing users to formulate queries and perform visualization, an Apache Web Service which facilitates the connection of the user application to the database and a relational database which is implemented using *MySQL *(Figure 1A). Utilization of the *Java WebStart *technology ensures that users will have the latest version of the application, without the need for manual updating. In addition, efficiency and speed of the application will largely be determined by the user's computer specifications. Hence, we have provided different versions of our application based on system resource utilization, allowing users with greater system resources to perform more analytical tasks per session.

### Database implementation and structure

*SIGMA *is a continually expanding database of array CGH experiments. The launch version contains 267 genomic profiles generated from cancer cell lines, implemented using the *MySQL *relational database application. Each array CGH experiment is contained in a separate database table allowing for easy and seamless expansion of this database. Upon addition of a new profile, a database table which contains the information of each array CGH experiment is updated. This table stores a record of each experiment, with the name of the cell line, the *American Type Culture Collection *(ATCC) identification (if applicable), array platform used and the description of the cancer type as part of the schema. For two channel array-based profiles, the dye which was used to label the sample is also recorded. Lastly, mapping information pertaining to a clone or probe and its position in the genome is kept in file with a fixed format, such that subsequent improvements and updates to the genomic positioning of the array elements can be easily incorporated. Moreover, since individual microarray software platforms use their own map version, map information for all platforms were compiled based on data from the *UCSC Genome Browser *[25]. Currently, two genomic builds are supported: April 2003 (hg15) and May 2004 (hg17) assemblies.

### Data processing

Data for each platform were processed as similarly as possible. SMRT array data were normalized using the stepwise framework for normalization with default parameters [26]. Similarly, Affymetrix 10 K and Affymetrix 100 K data were normalized and processed using *dChip *[27] with default settings. Specifically, the samples from the Affymetrix 10 K dataset of lung cancer cell lines were referenced against the group of matching blood lymphoblast lines and similarly, the breast cancer cell lines were referenced against their matching blood lymphoblast lines. Affymetrix 100 K data from Zhao *et al*. (2004) were referenced against 12 normal individuals and the NCI-60 profiles were referenced against 6 normal diploid controls. The gender of the profiles were not specified, hence data from the X chromosome may not be an accurate reflection. Segmentation of all data was performed using *aCGH-Smooth *[28], with data for the sex chromosomes removed prior to segmentation – as profiles were generated with sex matched or mismatched reference DNA in two channel hybridization experiments – and run with the settings of Lambda = 6.75 and "breakpoints per chromosome" = 100. Each element of the array is given a call with respect to normal: -1 if the element shows copy loss, 0 if the element shows no change in copy number and +1 if the element shows increased genomic content.

## Abbreviations

SIGMA: System for Integrative Genomic Microarray Analysis

CGH: Comparative Genomic Hybridization

SNP: Single Nucleotide Polymorphism

## Availability and requirements

Project name: SIGMA (System for Integrative Genomic Microarray Analysis)

Project home page: 

Operating system(s): Platform independent

Programming language: Java

Other requirements: Java version 1.6+

License: Free for academic and research use, commercial users please request special permission

## Authors' contributions

RC designed and developed SIGMA and wrote the manuscript. WWL and BPC were involved in the design and development. AC, DM and AT were the programmers. JJD was involved with initial design and CM provided manuscript editing and program feedback. WLL is the principal investigator of the lab.

## Supplementary Material

Additional file 1 List of cell lines in database. Detailed description of all the cell lines. Click here for file

Additional file 2 Single group visualization. Whole genome frequency plot of lung adenocarcinoma. Click here for file

Additional file 3 Two group whole genome comparison. Whole genome frequency plot comparison of squamous cancer of the lung and cervix. Click here for file

## Figures and Tables

**Table 1:** Breakdown of cell line sources by tumor type and study

**Tissue origin**	**Reference**	**# of samples**	**Array Platform**	**PubMed ID**	**Data Deposited Online?**	**Location**
Mantle cell lymphoma	de Leeuw *et al. (2004)*	8	SMRT Array	15229187	Yes	bccrc.ca/images/cg/wanlam_mcl_sd.txt
Non-small cell lung cancer	Garnis *et al. (2006)*	28	SMRT Array	16187286	Yes	GEO: GSE2922
Small cell lung cancer, Lymphoblast	Coe *et al.(2006a)*	21	SMRT Array	16130125	No	This study^2^
Breast Cancer	Shadeo *et al. (2006)*	7	SMRT Array	16417655	Yes	GEO: GSE3106
NCI-60 panel^1^	Garraway *et al. (2005)*	58	Affymetrix 100K SNP Array	16001072	Yes	GEO: GSE2520
Small cell lung cancer, Breast cancer, Lymphoblast	Zhao *et al. (2004)*	33	Affymetrix 10K SNP Array	15126342	Yes	research.dfci.harvard.edu/meyersonlab/snp/cellFile/
Non-small cell lung cancer	Zhao *et al. (2005)*	27	Affymetrix 100K SNP Array	15994928	Yes	research2.dfci.harvard.edu/dfci/snp/
Small cell lung cancer	Kim *et al. (2006)*	24	Stanford cDNA Array	16116477	Yes	genome-www5.stanford.edu/
Breast cancer	Bredel *et al. (2005)*	2	Stanford cDNA Array	15858140	Yes	genome-www5.stanford.edu/
Non-small cell lung cancer	Wilson *et al. (2006)*	2	MeDIP Array	16397413	No	This study^2^
Osteosarcoma	Lim *et al. (2004)*	1	SMRT Array	15660435	No	This study^2^
Assorted^3^	-	56	SMRT Array	-	-	This study^2^

^1^NCI-60 panel includes 58 of the 60 total cell lines.

^2^This study refers to previously unavailable data now publicly available in *SIGMA*

^3^Assorted tumor types including: Non-small cell lung cancer, small cell lung cancer, osteosarcoma, melanoma, hepatocellular carcinoma, cervical cancer, breast cancer, ovarian cancer, leukemia, and multiple myeloma

**Table 2:** Breakdown of cell lines based on tissue and array platform

**Tissue type**	**Distinct Cell Lines**	**Total Number**	**BCCRC SMRT**	**BCCRC MEDIP**	**Affymetrix 100K**	**Affymetrix 10K**	**Stanford cDNA**
Lung	74	123	54	2	36	7	24
Breast	22	37	17	0	7	11	2
Colon	8	9	2	0	7	0	0
Lymphoma	10	10	9	0	1	0	0
Leukemia	4	6	2	0	4	0	0
Brain/CNS	6	6	0	0	6	0	0
Lymphoblast^1^	20	31	16	0	0	15	0
Kidney	7	7	0	0	7	0	0
Skin	11	12	4	0	8	0	0
Cervix	8	8	8	0	0	0	0
Ovary	7	8	2	0	6	0	0
Prostate	3	5	3	0	2	0	0
Liver	1	1	1	0	0	0	0
Bone	2	2	2	0	0	0	0
Bone Marrow	1	2	1	0	1	0	0
**Grand Totals**	**184**	**267**	121	2	85	33	26

^1^Normal lymphoblast lines derived from patients with tumors

## References

[B1] Lockwood WW, Chari R, Chi B, Lam WL (2006). Recent advances in array comparative genomic hybridization technologies and their applications in human genetics. Eur J Hum Genet.

[B2] Davies JJ, Wilson IM, Lam WL (2005). Array CGH technologies and their applications to cancer genomes. Chromosome Res.

[B3] Pinkel D, Albertson DG (2005). Array comparative genomic hybridization and its applications in cancer. Nat Genet.

[B4] Barrett T, Suzek TO, Troup DB, Wilhite SE, Ngau WC, Ledoux P, Rudnev D, Lash AE, Fujibuchi W, Edgar R (2005). NCBI GEO: mining millions of expression profiles--database and tools. Nucleic Acids Res.

[B5] Parkinson H, Sarkans U, Shojatalab M, Abeygunawardena N, Contrino S, Coulson R, Farne A, Lara GG, Holloway E, Kapushesky M, Lilja P, Mukherjee G, Oezcimen A, Rayner T, Rocca-Serra P, Sharma A, Sansone S, Brazma A (2005). ArrayExpress--a public repository for microarray gene expression data at the EBI. Nucleic Acids Res.

[B6] La Rosa P, Viara E, Hupe P, Pierron G, Liva S, Neuvial P, Brito I, Lair S, Servant N, Robine N, Manie E, Brennetot C, Janoueix-Lerosey I, Raynal V, Gruel N, Rouveirol C, Stransky N, Stern MH, Delattre O, Aurias A, Radvanyi F, Barillot E (2006). VAMP: visualization and analysis of array-CGH, transcriptome and other molecular profiles. Bioinformatics.

[B7] Coe BP, Lockwood WW, Girard L, Chari R, Macaulay C, Lam S, Gazdar AF, Minna JD, Lam WL (2006). Differential disruption of cell cycle pathways in small cell and non-small cell lung cancer. Br J Cancer.

[B8] Garnis C, Lockwood WW, Vucic E, Ge Y, Girard L, Minna JD, Gazdar AF, Lam S, Macaulay C, Lam WL (2006). High resolution analysis of non-small cell lung cancer cell lines by whole genome tiling path array CGH. Int J Cancer.

[B9] Ching TT, Maunakea AK, Jun P, Hong C, Zardo G, Pinkel D, Albertson DG, Fridlyand J, Mao JH, Shchors K, Weiss WA, Costello JF (2005). Epigenome analyses using BAC microarrays identify evolutionary conservation of tissue-specific methylation of SHANK3. Nat Genet.

[B10] Weber M, Davies JJ, Wittig D, Oakeley EJ, Haase M, Lam WL, Schubeler D (2005). Chromosome-wide and promoter-specific analyses identify sites of differential DNA methylation in normal and transformed human cells. Nat Genet.

[B11] Khulan B, Thompson RF, Ye K, Fazzari MJ, Suzuki M, Stasiek E, Figueroa ME, Glass JL, Chen Q, Montagna C, Hatchwell E, Selzer RR, Richmond TA, Green RD, Melnick A, Greally JM (2006). Comparative isoschizomer profiling of cytosine methylation: The HELP assay. Genome Res.

[B12] Karimi M, Johansson S, Stach D, Corcoran M, Grander D, Schalling M, Bakalkin G, Lyko F, Larsson C, Ekstrom TJ (2006). LUMA (LUminometric Methylation Assay)--a high throughput method to the analysis of genomic DNA methylation. Exp Cell Res.

[B13] Wilson IM, Davies JJ, Weber M, Brown CJ, Alvarez CE, MacAulay C, Schubeler D, Lam WL (2006). Epigenomics: mapping the methylome. Cell Cycle.

[B14] Remmelink M, Mijatovic T, Gustin A, Mathieu A, Rombaut K, Kiss R, Salmon I, Decaestecker C (2005). Identification by means of cDNA microarray analyses of gene expression modifications in squamous non-small cell lung cancers as compared to normal bronchial epithelial tissue. Int J Oncol.

[B15] Zhao X, Li C, Paez JG, Chin K, Janne PA, Chen TH, Girard L, Minna J, Christiani D, Leo C, Gray JW, Sellers WR, Meyerson M (2004). An integrated view of copy number and allelic alterations in the cancer genome using single nucleotide polymorphism arrays. Cancer Res.

[B16] Garraway LA, Widlund HR, Rubin MA, Getz G, Berger AJ, Ramaswamy S, Beroukhim R, Milner DA, Granter SR, Du J, Lee C, Wagner SN, Li C, Golub TR, Rimm DL, Meyerson ML, Fisher DE, Sellers WR (2005). Integrative genomic analyses identify MITF as a lineage survival oncogene amplified in malignant melanoma. Nature.

[B17] de Leeuw RJ, Davies JJ, Rosenwald A, Bebb G, Gascoyne RD, Dyer MJ, Staudt LM, Martinez-Climent JA, Lam WL (2004). Comprehensive whole genome array CGH profiling of mantle cell lymphoma model genomes. Hum Mol Genet.

[B18] Coe BP, Lee EH, Chi B, Girard L, Minna JD, Gazdar AF, Lam S, Macaulay C, Lam WL (2006). Gain of a region on 7p22.3, containing MAD1L1, is the most frequent event in small-cell lung cancer cell lines. Genes Chromosomes Cancer.

[B19] Shadeo A, Lam WL (2006). Comprehensive copy number profiles of breast cancer cell model genomes. Breast Cancer Res.

[B20] Zhao X, Weir BA, LaFramboise T, Lin M, Beroukhim R, Garraway L, Beheshti J, Lee JC, Naoki K, Richards WG, Sugarbaker D, Chen F, Rubin MA, Janne PA, Girard L, Minna J, Christiani D, Li C, Sellers WR, Meyerson M (2005). Homozygous deletions and chromosome amplifications in human lung carcinomas revealed by single nucleotide polymorphism array analysis. Cancer Res.

[B21] Bredel M, Bredel C, Juric D, Kim Y, Vogel H, Harsh GR, Recht LD, Pollack JR, Sikic BI (2005). Amplification of whole tumor genomes and gene-by-gene mapping of genomic aberrations from limited sources of fresh-frozen and paraffin-embedded DNA. J Mol Diagn.

[B22] Kim YH, Girard L, Giacomini CP, Wang P, Hernandez-Boussard T, Tibshirani R, Minna JD, Pollack JR (2006). Combined microarray analysis of small cell lung cancer reveals altered apoptotic balance and distinct expression signatures of MYC family gene amplification. Oncogene.

[B23] Ball CA, Awad IA, Demeter J, Gollub J, Hebert JM, Hernandez-Boussard T, Jin H, Matese JC, Nitzberg M, Wymore F, Zachariah ZK, Brown PO, Sherlock G (2005). The Stanford Microarray Database accommodates additional microarray platforms and data formats. Nucleic Acids Res.

[B24] Ishkanian AS, Malloff CA, Watson SK, DeLeeuw RJ, Chi B, Coe BP, Snijders A, Albertson DG, Pinkel D, Marra MA, Ling V, MacAulay C, Lam WL (2004). A tiling resolution DNA microarray with complete coverage of the human genome. Nat Genet.

[B25] Hinrichs AS, Karolchik D, Baertsch R, Barber GP, Bejerano G, Clawson H, Diekhans M, Furey TS, Harte RA, Hsu F, Hillman-Jackson J, Kuhn RM, Pedersen JS, Pohl A, Raney BJ, Rosenbloom KR, Siepel A, Smith KE, Sugnet CW, Sultan-Qurraie A, Thomas DJ, Trumbower H, Weber RJ, Weirauch M, Zweig AS, Haussler D, Kent WJ (2006). The UCSC Genome Browser Database: update 2006. Nucleic Acids Res.

[B26] Khojasteh M, Lam WL, Ward RK, Macaulay C (2005). A stepwise framework for the normalization of array CGH data. BMC Bioinformatics.

[B27] Lin M, Wei LJ, Sellers WR, Lieberfarb M, Wong WH, Li C (2004). dChipSNP: significance curve and clustering of SNP-array-based loss-of-heterozygosity data. Bioinformatics.

[B28] Jong K, Marchiori E, Meijer G, Vaart AV, Ylstra B (2004). Breakpoint identification and smoothing of array comparative genomic hybridization data. Bioinformatics.

