# Supplementary material for: SIGMA: A System for Integrative Genomic Microarray Analysis of Cancer Genomes
Source: BMC Genomics. 2006 Dec 27;7:324. doi: 10.1186/1471-2164-7-324 (PMC1764892; doi:10.1186/1471-2164-7-324)
Supplement: Additional file 2 — Single group visualization. Whole genome frequency plot of lung adenocarcinoma. [file 1471-2164-7-324-S2.pdf]

Additional file 2: Single group visualization

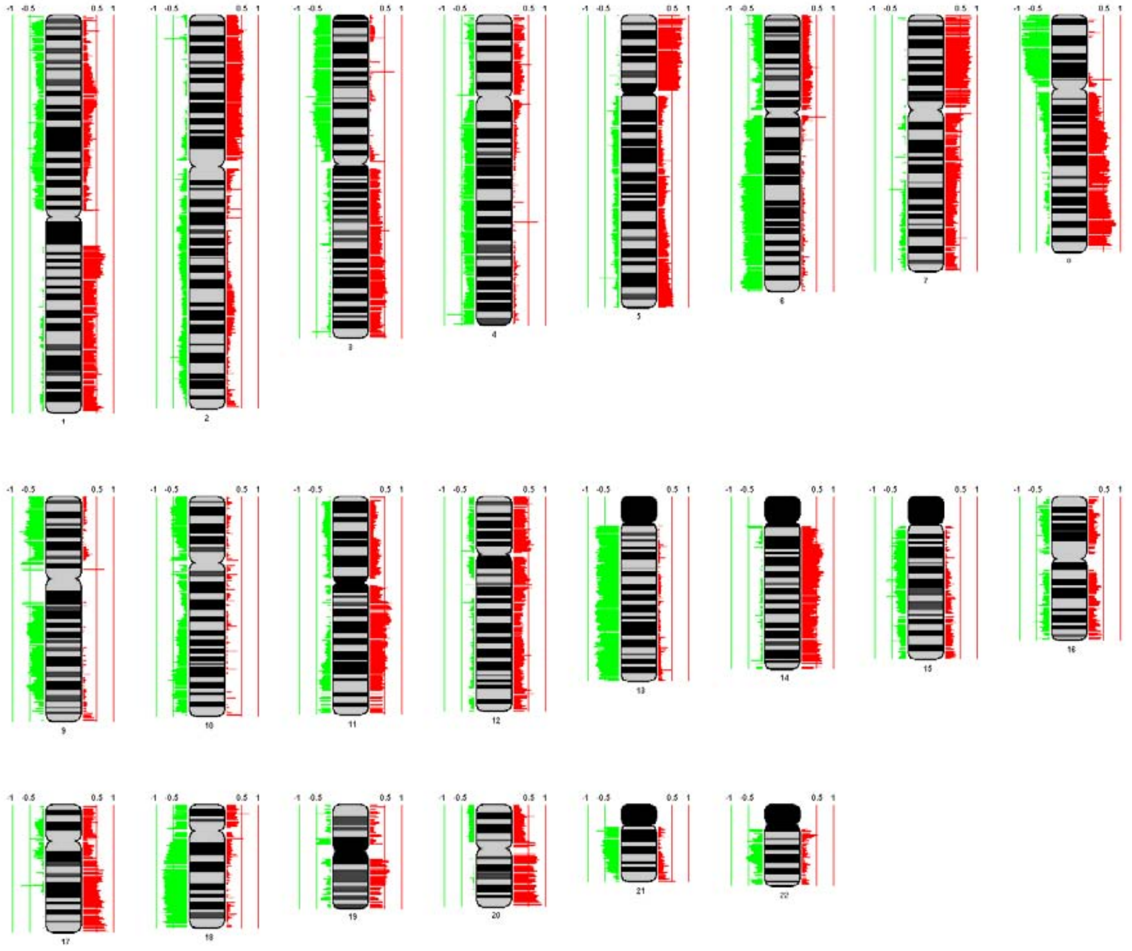

Additional file 2. Whole genome frequency plot analysis of 24 lung adenocarcinoma samples profiled on the SMRT array using the May 2004 genomic build. Regions colored in green denote areas of recurrent loss, with the maximum value of -1 representing loss of that area in all samples. Similarly, regions colored in red denote areas of recurrent gain, with the maximum value of 1 representing gain of that area in all samples.
