# Supplementary material for: SIGMA: A System for Integrative Genomic Microarray Analysis of Cancer Genomes
Source: BMC Genomics. 2006 Dec 27;7:324. doi: 10.1186/1471-2164-7-324 (PMC1764892; doi:10.1186/1471-2164-7-324)
Supplement: Additional file 3 — Two group whole genome comparison. Whole genome frequency plot comparison of squamous cancer of the lung and cervix. [file 1471-2164-7-324-S3.pdf]

Additional file 3: Two group whole genome comparison

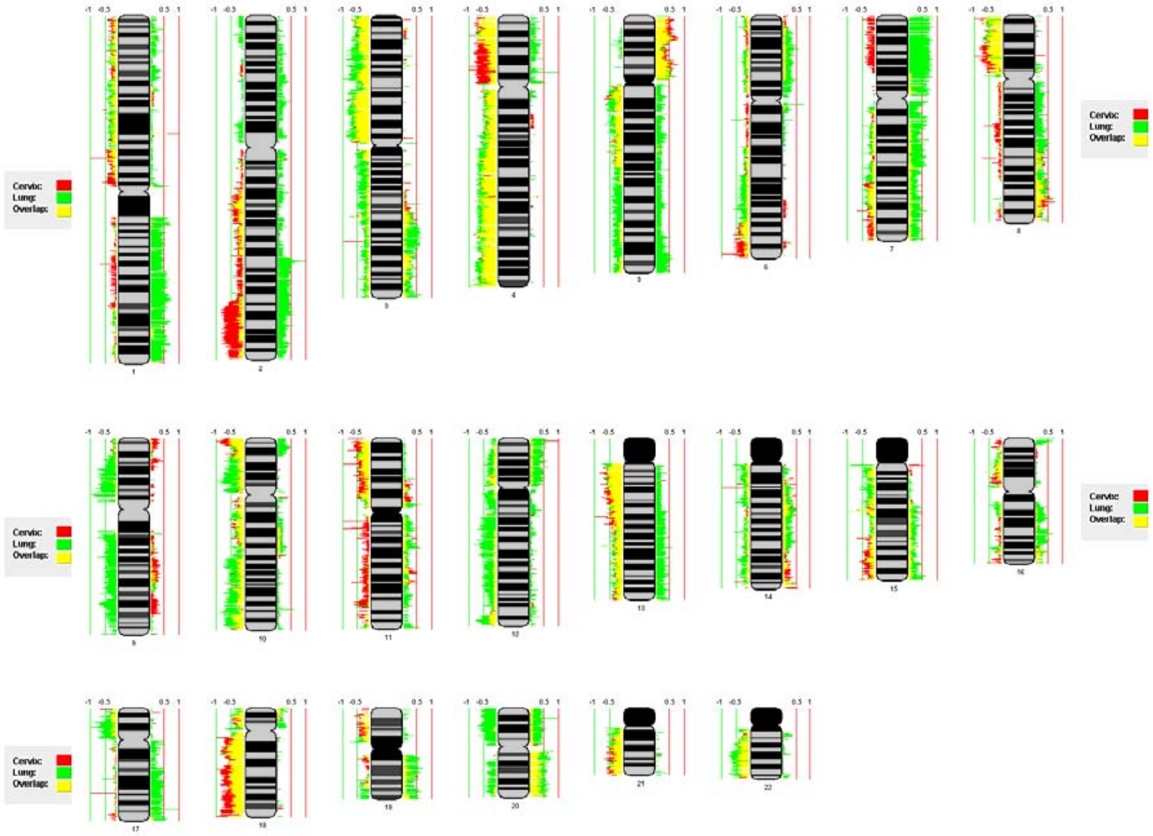

Additional file 3. Overlay of two whole genome frequency plots representing 9 lung squamous cancer cell lines (red) and 7 cervical squamous cancer cell lines (green), with common regions of recurrence in yellow.
